# Association of Race, Ethnicity, and Rurality With Major Leg Amputation or Death Among Medicare Beneficiaries Hospitalized With Diabetic Foot Ulcers

**DOI:** 10.1001/jamanetworkopen.2022.8399

**Published:** 2022-04-01

**Authors:** Meghan B. Brennan, W. Ryan Powell, Farah Kaiksow, Joseph Kramer, Yao Liu, Amy J. H. Kind, Christie M. Bartels

**Affiliations:** Department of Medicine, University of Wisconsin, Madison; Department of Medicine, University of Wisconsin, Madison; Department of Medicine, University of Wisconsin, Madison; Department of Medicine, University of Wisconsin, Madison; Department of Ophthalmology, University of Wisconsin, Madison; Department of Medicine, University of Wisconsin, Madison; Center for Health Disparities Research, University of Wisconsin School of Medicine and Public Health, Madison; Geriatric Research Education and Clinical Center (GRECC), William S. Middleton Hospital, Department of Veterans Affairs, Madison, Wisconsin; Department of Medicine, University of Wisconsin, Madison

## Abstract

**IMPORTANCE:**

Patients identifying as Black and those living in rural and disadvantaged neighborhoods are at increased risk of major (above-ankle) leg amputations owing to diabetic foot ulcers. Intersectionality emphasizes that the disparities faced by multiply marginalized people (eg, rural US individuals identifying as Black) are greater than the sum of each individual disparity.

**OBJECTIVE:**

To assess whether intersecting identities of Black race, ethnicity, rural residence, or living in a disadvantaged neighborhood are associated with increased risk in major leg amputation or death among Medicare beneficiaries hospitalized with diabetic foot ulcers.

**DESIGN, SETTING, AND PARTICIPANTS:**

This retrospective cohort study used 2013-2014 data from the US National Medicare Claims Data Database on all adult Medicare patients hospitalized with a diabetic foot ulcer. Statistical analysis was conducted from August 1 to October 27, 2021.

**EXPOSURES:**

Race was categorized using Research Triangle Institute variables. Ruralitywas assigned using Rural-Urban Commuting Area codes. Residents of disadvantaged neighborhoods comprised those living in neighborhoods at or above the national 80th percentile Area Deprivation Index.

**MAIN OUTCOMES AND MEASURES:**

Major leg amputation or death during hospitalization or within 30 days of hospital discharge. Logistic regression was used to explore interactions among race, ethnicity, rurality, and neighborhood disadvantage, controlling for sociodemographic characteristics, comorbidities, and ulcer severity.

**RESULTS:**

The cohort included 124 487 patients, with a mean (SD) age of 71.5 (13.0) years, of whom 71 286 (57.3%) were men, 13 100 (10.5%) were rural, and 21 649 (17.4%) identified as Black. Overall, 17.6% of the cohort (n = 21 919), 18.3% of rural patients (2402 of 13 100), and 21.9% of patients identifying as Black (4732 of 21 649) underwent major leg amputation or died. Among 1239 rural patients identifying as Black, this proportion was 28.0% (n = 347). This proportion exceeded the expected excess for rural patients (18.3% − 17.6% = 0.7%) plus those identifying as Black (21.9% − 17.6% = 4.3%) by more than 2-fold (28.0% − 17.6% = 10.4% vs 0.7% + 4.3% = 5.0%). The adjusted predicted probability of major leg amputation or death remained high at 24.7% (95% CI, 22.4%-26.9%), with a significant interaction between race and rurality.

**CONCLUSIONS AND RELEVANCE:**

Rural patients identifying as Black had a more than 10% absolute increased risk of major leg amputation or death compared with the overall cohort. This study suggests that racial and rural disparities interacted, amplifying risk. Findings support using an intersectionality lens to investigate and address disparities in major leg amputation and mortality for patients with diabetic foot ulcers.

## Introduction

Approximately 5% of individuals in the US with diabetic foot ulcers require a major (ie, above-ankle) leg amputation.^1^ Many fear major leg amputation more than death,^2^ and the Agency for Healthcare Research and Quality listed amputation as 1 of 16 “never-event” quality indicators.^3,4^

In the US, individuals share the burden of major leg amputations unequally.^5^ Patients identifying as Black undergo amputation at twice the rate of those identifying as non-Hispanic White.^6^ Rural patients have approximately 35% higher odds of major leg amputation vs urban counterparts.^7,8^

Neighborhood disadvantage can be quantified using the Area Deprivation Index (ADI), a multidomain of neighborhood disadvantage constructed at the Census Block Group level from 17 measures of housing quality, income, educational level, and employment.^9^ In Europe, neighborhood disadvantage has been associated with greater risk of major leg amputation owing to diabetic foot ulcers.^10,11^ In the US, neighborhood disadvantage has been associated with poor health care quality and diabetes outcomes.^12^

Although racial, rural, and neighborhood disadvantage are well established, likely reflecting historic and ongoing systems of marginalization and oppression, we do not know how these social identities coalesce to affect diabetic foot ulcer outcomes.^13^ Intersectionality is a theoretical framework rooted in Black feminist legal studies with a goal of improving social justice for multiply marginalized people, such as rural US individuals identifying as Black.^14^ An essential principle of intersectionality is that the effects of different, interlocking disadvantages amplify in a matrix of oppresion.^15-17^ Core tenets of intersectionality include overlapping identities, historically oppressed populations, and social determinants of health.^18^ We hypothesized that rural patients identifying as Black face an amplified risk of major leg amputation or death. Prior studies have demonstrated that (1) rural US counties where most residents identify as Black have the highest rates of all-cause premature death^19^ and (2) individuals identifying as Black and living in rural US counties face amplified odds of poor outcomes owing to peripheral vascular disease and cancer.^20,21^ This study aims to describe how intersectionality among race, ethnicity, rurality, and neighborhood disadvantage may be associated with risks of major leg amputations and death among Medicare beneficiaries hospitalized with diabetic foot ulcers. This study is a preceding step to analytic intersectionality, which aims to identify and rectify causal processes.^22^

## Methods

### Data Sources

We used a 100% national sample of adult, traditional Medicare beneficiaries during acute hospitalization (from January 1, 2013, to December 31, 2014, from the US National Medicare Claims Data Database). We required patients to be continuously enrolled in Medicare Parts A and B for 12 months preceding the index hospitalization and to have a linkable address. We used patient 9-digit zip codes to link Medicare data to categorize rurality (Rural-Urban Commuting Area [RUCA] codes) and neighborhood disadvantage (Neighborhood Atlas ADI).^23,24^ The University of Wisconsin health sciences institutional review board approved this study and waived written informed consent because the data set was deidentified. Findings are reported per the Strengthening the Reporting of Observational Studies in Epidemiology (STROBE) reporting guideline.^25^

### Study Design

We constructed and analyzed a retrospective national cohort of Medicare beneficiaries hospitalized with diabetic foot ulcers by first identifying patients with diabetes based on the Chronic Conditions Data Warehouse category.^26^ Next, we categorized diabetic foot ulcers as early stage, osteomyelitis, or gangrene using a validated algorithm.^27^ We excluded patients with health maintenance organization or railroad benefits owing to incomplete claims.^28^ For patients hospitalized more than once, only the first index admission was included. We followed up with patients for 30 days after hospital discharge to assess outcomes.

### Primary Composite Outcome

Our outcome was a composite of major leg amputation or death during the index hospitalization or within 30 days after hospital discharge. We used *International Classification of Diseases, Ninth Revision* codes and *Current Procedural Terminology* codes to identify major leg amputation (eTable 1 in the Supplement).^1^ More than 99% of death dates reported in Medicare data are confirmed using a Medicare Common Working File with information submitted directly by family or the Social Security Administration.^29^

### Primary Explanatory Variables

We investigated 3 social identitymetrics: race, ethnicity, rurality, and neighborhood disadvantage. We used an intercategorical approach, assuming that social identities can be categorized.^30^ The Research Triangle Institute race variable used an algorithm drawing on Medicare enrollment forms, Social Security registration, and beneficiary surveys wherein patients identify as American Indian or Alaska Native, Asian or Pacific Islander, Black or African American (hereafter referred to as Black), Hispanic, other (a designation that beneficiaries could select without providing further details), unknown, and non-Hispanic White (hereafter referred to as White).^31,32^ The RUCA codes were used to assign rurality and rural-urban commuting gradients: urban (RUCA 1 [reference]), suburban (RUCA 2-6), and rural (RUCA 7-10).^23^ Neighborhood disadvantage was measured using ADI. More disadvantaged neighborhoods receive higher ADI scores. We dichotomized neighborhood disadvantage based on those living below (reference) or above the national 80th percentile ADI (most disadvantaged) based on published ADI thresholds in which the 80th percentile was associated with worse outcomes.^9^

### Covariates

Sociodemographic variables included age, sex, and receipt of Medicaid coverage the year prior to hospitalization. We adjusted for ulcer severity and comorbidities using the validated ulcer algorithm, the baseline-year Hierarchical Condition Category, Medicare Chronic Conditions Data Warehouse categories, and Elixhauser Comorbidity Index variables.^27^ Higher Hierarchical Condition Category scores correlate with higher health care costs and use.^33,34^ The following comorbidities were identified using validated Medicare Chronic Conditions Data Warehouse conditional categories: myocardial infarction, ischemic heart disease, hyperlipidemia, hypertension, and stroke.^35^ The following Elixhauser Comorbidity Index comorbidities with less than 5% prevalence in our cohort were consolidated into a single indicator variable (considered positive if any of the 9 comorbidities were present): AIDS or HIV, alcohol use disorder, blood loss anemia, drug use disorder, lymphoma, metastatic cancer, peptic ulcer disease without bleeding, rheumatoid arthritis or collagen vascular disease, and solid tumor without metastasis.^36^

### Statistical Analysis

Statistical analysis was conducted from August 1 to October 27, 2021. We described patient characteristics, both overall and stratified by race. Because we used a full 100% hospitalized Medicare patient data set, descriptive statistics measure actual differences between subgroups, not estimates. We relied primarily on observed differences to assess for intersectionality because of this strength. However, we also used statistical modeling based on interaction terms to explore intersectionality on the additive and multiplicative scales.^37,38^

We performed blockwise logistic regression to assess how covariates and interactions influenced the associations between our social identitymetrics and major leg amputation or death among Medicare beneficiaries hospitalized with diabetic foot ulcers.^39^ We excluded patients with incomplete data during statistical analysis. When examining race, we report regression results only for patients identifying as Black compared with White owing to concerns that the Research Triangle Institute race variable may miscategorize patients identifying as other races and because there are low numbers of rural patients identifying as Hispanic (n = 431), limiting interpretation for our primary rural focus.^32^ For all models, the dependent variable was major leg amputation or death during the index hospitalization or within 30 days of hospital discharge. First, we separately analyzed associations between each of our social identitymetrics (identifying as Black, rural residence, and living in a disadvantaged neighborhood, each in their own model) and death or major leg amputation, controlling for sex and age. Second, we built a single model including all 3 social identitymetrics and adjusting for all of our sociodemographic characteristics. Third, we built a model with all 3 social identitymetrics, sociodemographic covariates, ulcer severity covariates, and comorbidity covariates. Fourth, we tested interaction terms among rurality, identification as Black, and neighborhood disadvantage, including a 3-way interaction. Interaction terms with 2-sided *P* < .001 met our a priori level of statistical significance.

Odds ratios (ORs) associated with social identitymetrics were interpreted as the association of a metric after controlling for other factors that may be on the causal pathway to the outcome on a multiplicative scale.^40^ For instance, more prevalent comorbidities are likely on the causal pathway between identifying as Black and our outcome. Likewise, age and sex might be associated with outcomes through social factors, such as driving.^41,42^ When associations between identifying as Black and major leg amputation or death were modeled, controlling for comorbidities, ORs should be interpreted as relative differences that would remain if comorbidities were held constant across patients identifying as Black or White. Marginal standardized methods, standardized to the overall cohort, were used to estimate predicted probabilities of major leg amputation and death and to assess for intersectionality on an additive scale, which best informs policy.^37,43^ Both predicted and observed proportions are presented. We used STATA, version 17 software (StataCorp LLC) to perform our statistical analysis.^44^

## Results

During the 2-year study period, 124 487 Medicare beneficiaries (71 286 men [57.3%]; mean [SD] age, 71.5 [13.0]; 13 100 rural residents [10.5%]) were hospitalized with diabetic foot ulcers (Table 1). A total of 21 649 individuals (17.4%) identified as Black, 10 158 (8.2%) identified as Hispanic, 88 525 (71.1%) identified as White, and 4155 (3.3%) identified as another race or ethnicity (American Indian or Alaska Native, Asian or Pacific Islander, other races or ethnicities, and unknown race and ethnicity). Overall, 13 451 patients (10.8%) died and 9617 patients (7.7%) underwent a major leg amputation during the index hospitalization or within 30 days of hospital discharge.

Compared with patients identifying as White, a higher proportion of patients in racial and ethnic minority groups lived in urban and disadvantaged neighborhoods (Table 1). Patients identifying as part of a racial and ethnic minority group tended to be younger than those identifying as White, but they also tended to experience higher proportions of comorbidities, including stroke and transient ischemic events, peripheral vascular disease, and kidney failure. In particular, there was a nearly 2-fold difference among those presenting with gangrenous ulcers between patients identifying as Black and patients identifying as White (4428 of 21 649 [20.5%] vs 9331 of 88 525 [10.5%]).

**Figure 1.**
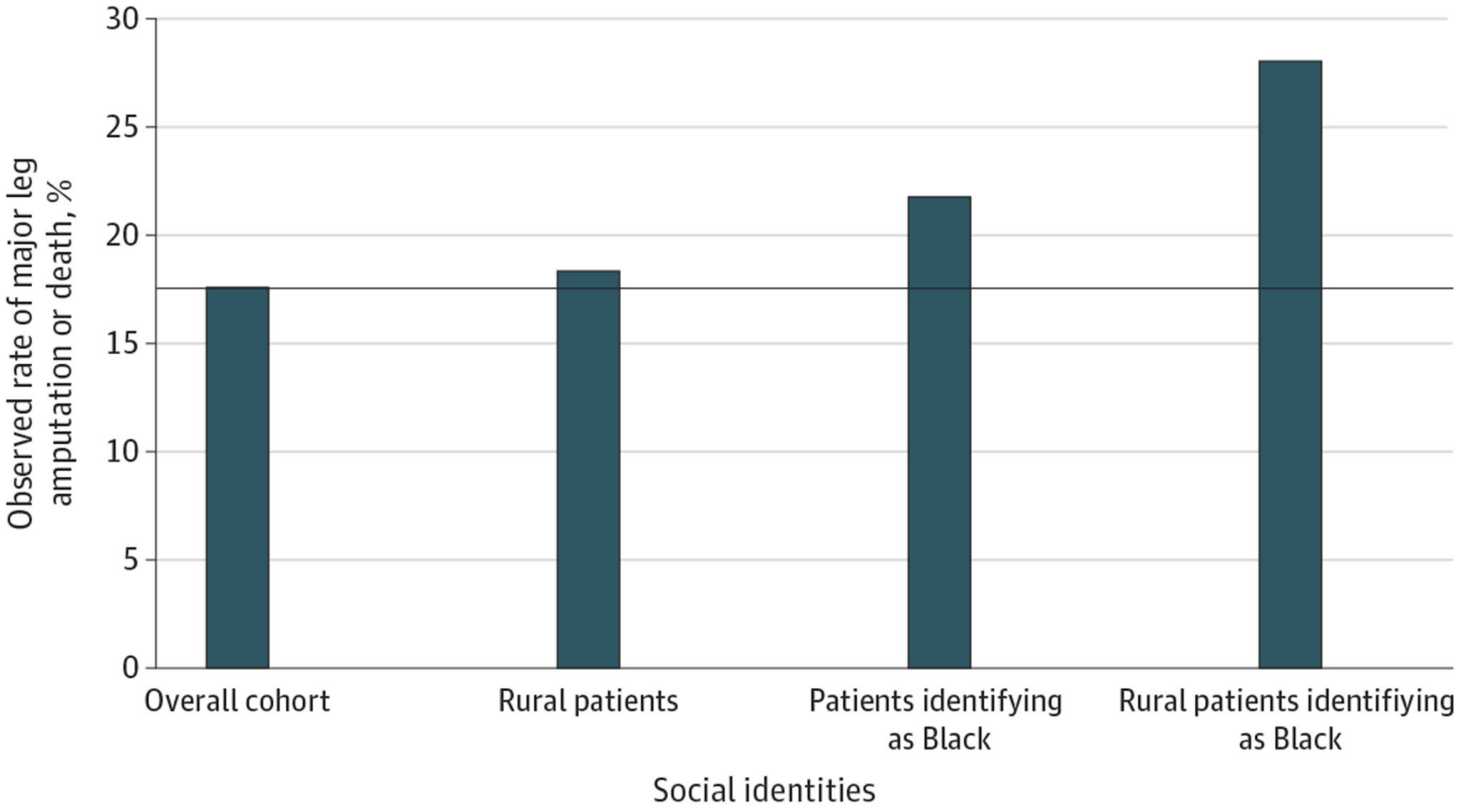
Observed Rates of Major Leg Amputation or Death Observed rates of major (above-ankle) leg amputation or death among the full cohort, rural patients, patients identifying as Black, and rural patients identifying as Black. The horizontal line marks the observed rate in the overall cohort, 17.6%. Observed excess morbidity and mortality are depicted above this line for each marginalized social identity.

Patients identifying as Black who were hospitalized with diabetic foot ulcers had a 21.9% rate of death or major leg amputation (4732 of 21 649), an absolute percentage point increase of 4.3 compared with the overall cohort’s proportion of 17.6%(n = 21 919) (Figure 1). The observed proportion of death or major leg amputation for rural patients was 18.3%(2402 of 13 100), an increase of 0.7 percentage points (Figure 1; Table 2). The proportion of major leg amputation or death among rural patients identifying as Black was 28.0%(347 of 1239), more than 10% higher than the overall cohort (Figure 1). Using a multiple, not intersectional, approach, one might assume that the disparity experienced by rural patients also identifying as Black could be estimated by summing excess morbidity and mortality associated with disaggregated identities (0.7% for rural excess + 4.3% for identifying as Black excess = 5.0%).^45,46^ However, the observed excess was 10.4%(28.0% for observed rural patients identifying as Black − 17.6% for observed overall = 10.4%), consistent with intersectionality. Last, a higher proportion of patients living in disadvantaged neighborhoods underwent major leg amputation or died compared with those in nondisadvantaged neighborhoods (5025 of 26 430 [19.0%] vs 15 826 of 92 045 [17.2%]) (Table 2).

Our statistical models support a role for intersectionality among rural patients identifying as Black on the multiplicative scale, reported as ORs (Table 2 and Table 3). We found a significant interaction between identifying as Black and rural residence (OR, 1.34; 95% CI, 1.15-1.57), indicating that the associations of these 2 social identities were amplified (Table 3, model 4). The odds of major leg amputation or death increased 14% for rural patients identifying as White compared with urban patients identifying as White (OR, 1.14; 95% CI, 1.06-1.22). However, the odds increased by more than 80% for rural patients identifying as Black compared with urban patients identifying as Black (OR, 1.88; 95% CI, 1.41-2.50; interaction term between rural residence and identifying as Black: OR, 1.34; 95% CI, 1.15-1.57; *P* < .001). No other statistically significant interaction terms were found (eTable 2 in the Supplement).

**Figure 2.**
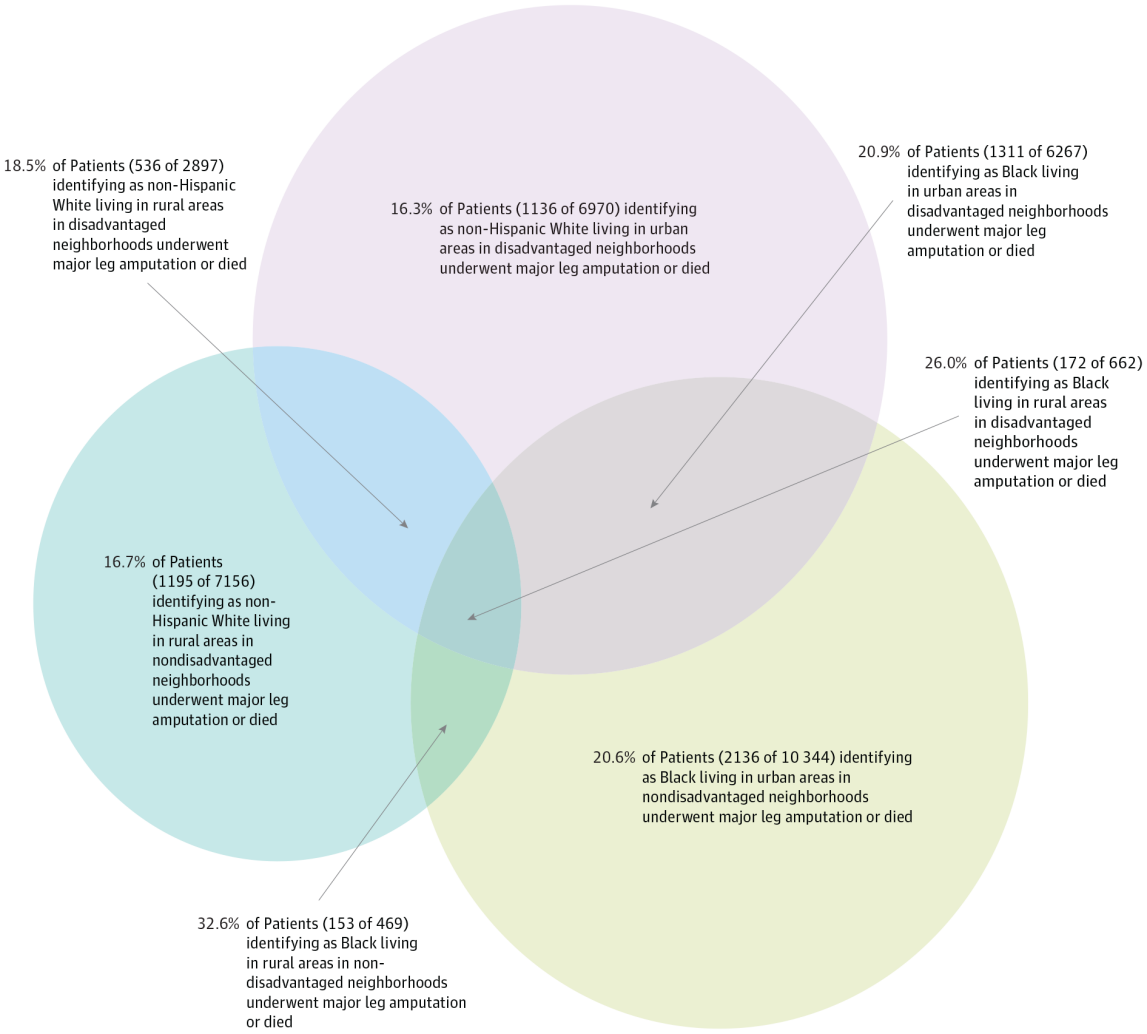
Intersections Between Social IdentityMetrics Intersections between our 3 social identitymetrics: identifying as Black, rural residence, and living in a disadvantaged neighborhood. Observed rates of major (above-ankle) leg amputation or death during hospitalization or within 30 days of hospital discharge are presented for the complete population of Medicare patients hospitalized with a diabetic foot ulcer. The overall observed rate in the cohort was 17.6%. This proportional Venn diagram was made using R software (R Project for Statistical Computing).^47^

We also found evidence for intersectionality on the additive scale using adjusted predicted probabilities, which may hold the most relevance for policy change (Table 2; Figure 2).^47^ Overall, 19.4%(95% CI, 18.9%-20.0%) of patients identifying as Black who were hospitalized with diabetic foot ulcers were predicted to undergo major leg amputation or die compared with 17.3%(95% CI, 17.0%-17.6%) of patients identifying as White. Among rural patients identifying as Black, this estimate increased to 24.7%(CI, 22.4%-26.9%), controlling for age, sex, comorbidities, and other socioeconomic variables.

## Discussion

We observed significantly higher rates of major leg amputation or death among all patients identifying as Black compared with the overall cohort. In particular, rural patients identifying as Black had an observed 28.1% risk of undergoing a major leg amputation or death after admission for a diabetic foot ulcer. This finding was more than 10 absolute percentage points higher than the overall national cohort. We assert that these differences are clinically and socially meaningful, particularly given the use of a complete Medicare population, which eliminates selection bias and ensures internal validity. We urge using an intersectionality approach to address this disparity.

This study highlights the intersectionality between identifying as Black and rural residence. Rural patients identifying as Black may face an amplified risk of death or major leg amputation after admission for a diabetic foot ulcer as evidenced by our final model’s significant interaction term. Our findings demonstrate that the forces underlying racial and rural disparities interact to worsen the health of multiply marginalized individuals. Thus, an intersectionality lens is critical to investigate and address the disparities faced by rural US individuals identifying as Black, including dismantling structural forces that perpetuate inequities.^48,49^

Our findings suggest that the broadly defined health care system may be one of the structural forces associated with the disparities in major leg amputations. The association between rurality and major leg amputation increased after controlling for comorbidity. This finding suggests that factors beyond comorbidities are associated with the increased odds of major leg amputation or death that we observed among rural patients. One such factor may be the eroding rural health care infrastructure.^50^ This infrastructure includes limited rural access to specialists, facility closures, and an understaffed workforce.^51,52^ Nearly 65% of rural counties are areas with a health professional shortage.^53^ This percentage increases to 83% among rural counties where most residents identify as Black. Deficits specific to foot ulcer care have also been documented, including limited access to vascular surgeons and infectious disease specialists in rural areas.^7,54,55^ Most multidisciplinary limb salvage teams operate in urban tertiary care centers.^56^ Rural patients are likely to have limited access to such teams in the absence of standardized triage systems.^57^ Improving triage across rural and urban health systems may be particularly effective for rural patients identifying as Black who develop diabetic foot ulcers. In addition, we report that patients identifying as Black are hospitalized with gangrenous foot ulcers at nearly twice the rate as those identifying as White. This level of ulcer severity suggests delayed or poor ambulatory care. Focusing efforts to improve care further upstream in ambulatory—as opposed to hospital—settings will likely prove to be particularly effective.

The associations between intersecting disparities are likely underrecognized given the tendency to think across a single-axis framework.^14^ Using only a race-based or rurality-based approach would miss the amplified associations that we report when these 2 social identities overlap. Multiaxis frameworks may help to identify and address the associations of interacting social identities. For instance, the National Institute on Minority Health and Health Disparities Research Framework offers a matrix of levels and domains that are associated with health outcomes.^58^ A goal of presenting the framework as a matrix was to account for interacting factors, such as race, ethnicity, and rurality. Authors cautioned that conducting research entirely within 1 cell of the matrix, without addressing the cumulative or interactive associations of multiple determinants, risks arriving at an incomplete understanding of a health phenomenon. Moving forward, using multiaxis frameworks to construct interventions aimed at improving disparities, especially those experienced by multiply marginalized populations, may be particularly useful.

To our knowledge, ours is the first study using a US population to establish an association between neighborhood disadvantage and risk of major leg amputation or death due to diabetic foot ulcers. This finding extends earlier work from the United Kingdom.^10,11^ A prior study of patients in Johns Hopkins University's multidisciplinary clinic found no association between neighborhood deprivation and major leg amputation, raising the possibility that such teams may help mitigate disparities.^59^ This approach hinges on the ability of disadvantaged populations to have access to these multidisciplinary teams.

Our model conservatively estimates differences across our 3 social identitymetrics. Adjusted, predicted probability rates for patients identifying as Black by place (rurality and neighborhood disadvantage) were below observed rates. Our model is conservative given that we controlled for measured comorbidities that likely are on the causal pathway between social identity and outcomes, such as peripheral vascular disease and ulcer severity. Therefore, the ORs in our final model should be interpreted as the residual disparity that would remain after all other measurable covariate factors were equally distributed across groups.^40^ Even within the bounds of these conservative models, we found significant differences across our social identitymetrics, suggesting that interventions targeting structural forces are necessary.

### Limitations

Despite the strengths of using a 100% national Medicare population, we acknowledge limitations to our study, especially regarding generalization. First, Medicare claims data are composed largely of older adults and capture an insured population. The recent resurgence in major leg amputations owing to diabetic foot ulcers is most pronounced in the US among individuals younger than 65 years, a demographic population underrepresented in our cohort.^60^ Furthermore, those without insurance are at increased risk of major leg amputation.^8,61^ In the US, lack of insurance is a critical issue for rural individuals identifying as Black, who are uninsured at higher rates than either urban individuals identifying as Black or rural individuals identifying as White.^53^ Our data set spanned from 2013 to 2014 and may not reflect current trends. However, we would counter that the rural mortality gap, both overall and due to diabetes, is expanding.^50,62^ Therefore, we may have underestimated current disparities. Limitations regarding internal validity include that claims data may introduce misclassification or may underestimate comorbidities, especially vascular disease. Gaps may be larger for marginalized patients interacting less with health care systems.^1^ Our multivariable analysis focused on rural individuals identifying as Black owing to concerns about miscategorizing patients identifying as being a part of other racial and ethnic minority groups. The experiences and the history of these minority populations living in rural areas in the US are unique. Moving forward, we suggest studies of younger populations, those spanning the full spectrum of insurance coverage, and rural people identifying as being part of other racial and ethnic groups. We need to move from descriptive to analytic intersectionality, which aims to address causal process, thereby improving social justice.^22^ Our results suggest that interventions (eg, investing in rural health care infrastructure and building connections between rural clinics and urban specialty centers) aimed at structural inequities, including those discussed and rooted in the US health care system, are necessary.

## Conclusions

In this cohort study, rural patients identifying as Black had a greater than 10 absolute percentage point increase in the rate of major amputation or death compared with the overall cohort. Racial and rural disparities interact, yielding an amplified risk of this outcome. Our findings support using an intersectionality lens to address disparities among patients with diabetic foot ulcers.

## Supplementary Material

Online Only Supplement Material **eTable 1.** *ICD-9* and *CPT* Procedure Codes Used to Identify Patients Undergoing Major Leg Amputation
**eTable 2.** Odds Ratios for Major Leg Amputation or Death for Main Effects and Interactions Among Social Identities of Interest: Identifying as Black, Rural Residence, and Neighborhood Disadvantage

## Figures and Tables

**Table 1.** Characteristics of Medicare Beneficiaries Hospitalized With Diabetic Foot Ulcers by Race and Ethnicity

Characteristic	Medicare beneficiaries, No. (%)				
	Entire cohort (N = 124 487)	Patients identifying as Black (n = 21 649)	Patients identifying as Hispanic (n = 10 158)	Patients identifying as White (n = 88 525)	Patients identifying as another race or ethnicity (n = 4155)^a^
Outcomes					
Death during index hospitalization or ≤30 d of discharge	13 451 (10.8)	2273 (10.5)	957 (9.4)	9789 (11.1)	432 (10.4)
Major amputation during index hospitalization or ≤30 d of discharge	9617 (7.7)	2779 (12.8)	1021 (10.1)	5458 (6.2)	359 (8.6)
Composite major (above-ankle) leg amputation or death during index hospitalization or ≤30 d of discharge	21 919 (17.6)	4732 (21.9)	1856 (18.3)	14 576 (16.5)	755 (18.2)
Baseline characteristics					
Age, mean (SD), y	71.5 (13.0)	68.2 (14.1)	68.3 (13.5)	72.9 (12.4)	68.5 (13.1)
Sex					
Female	53 201 (42.7)	10 386 (48.0)	4096 (40.3)	37 078 (41.9)	1641 (39.5)
Male	71 286 (57.3)	11 263 (52.0)	6062 (59.7)	51 447 (58.1)	2514 (60.5)
Rurality					
Urban	84 590 (68.0)	17 346 (80.1)	8075 (79.5)	56 585 (63.9)	2584 (62.2)
Suburban	26 262 (21.1)	3003 (13.9)	1283 (12.6)	21 175 (23.9)	801 (19.3)
Rural	13 100 (10.5)	1239 (5.7)	431 (4.2)	10 707 (12.1)	723 (17.4)
Unknown	535 (0.4)	61 (0.3)	369 (3.6)	58 (0.1)	47 (1.1)
Reside in ≥80th percentile ADI	26 430 (21.2)	8252 (38.1)	3219 (31.7)	14 111 (15.9)	848 (20.4)
Unknown ADI percentile	6012 (4.8)	1145 (5.3)	583 (5.7)	3847 (4.4)	437 (10.5)
Ever received Medicaid benefits	46 199 (37.1)	11 929 (55.1)	6570 (64.7)	25 551 (28.9)	2149 (51.7)
Ulcer severity					
Early stage	81 277 (65.3)	12 735 (58.8)	5823 (57.3)	60 227 (68.0)	2492 (60.0)
Osteomyelitis	26 892 (21.6)	4486 (20.7)	2432 (23.9)	18 967 (21.4)	1007 (24.2)
Gangrene	16 318 (13.1)	4428 (20.5)	1903 (18.7)	9331 (10.5)	656 (15.8)
History of myocardial infarction	22 768 (18.3)	3510 (16.2)	1898 (18.7)	16 635 (18.8)	725 (17.5)
History of ischemic heart disease	104 863 (84.2)	18 209 (84.1)	8710 (85.8)	74 645 (84.3)	3299 (79.4)
History of stroke or TIA	40 772 (32.8)	8571 (39.6)	3452 (34.0)	27 557 (31.1)	1192 (28.7)
History of hyperlipidemia	116 144 (93.3)	19 895 (91.9)	9593 (94.4)	82 855 (93.6)	3801 (91.5)
History of hypertension	123 368 (99.1)	21 575 (99.7)	10 079 (99.2)	87 612 (99.0)	4102 (98.7)
History of obesity	29 370 (23.6)	4999 (23.1)	1998 (19.7)	21 578 (24.4)	795 (19.1)
History of peripheral vascular disease	48 077 (38.6)	9313 (43.0)	4436 (43.7)	32 813 (37.1)	1515 (36.5)
History of kidney failure	51 841 (41.6)	11 786 (54.4)	5182 (51.0)	32 941 (37.2)	1932 (46.5)
HCC community score, mean (SD)	2.68 (1.90)	2.98 (2.06)	2.74 (1.84)	2.59 (1.85)	2.66 (1.89)

Abbreviations: ADI, Area Deprivation Index; HCC, Hierarchical Condition Category; TIA, transient ischemic attack.

a Includes 1481 patients identifying as American Indian or Alaska Native, 1368 patients identifying as Asian or Pacific Islander, 832 patients identifying as other races or ethnicities, and 474 patients whose race and ethnicity are unknown.

**Table 2.** Observed Proportions and Adjusted Predicted Probabilities of Major Leg Amputation or Death

Characteristic	No.	Observed proportions, No. (%)	Adjusted predicted probabilities, % (95% CI)^a^
Population			
Entire cohort	124 487	21 919 (17.6)	NA
Patients identifying as Black	21 649	4732 (21.9)	19.4 (18.9-20.0)
Patients identifying as White	88 525	14 576 (16.5)	17.3 (17.0-17.6)
Urban residents	84 590	14 620 (17.3)	16.9 (16.7-17.2)
Suburban residents	26 262	4789 (18.2)	19.2 (18.7-19.7)
Rural residents	13 100	2402 (18.3)	19.8 (19.1-20.6)
Nondisadvantaged neighborhoods	92 045	15 826 (17.2)	17.4 (17.1-17.6)
Disadvantaged neighborhoods	26 430	5025 (19.0)	18.6 (18.1-19.1)
Patients identifying as Black by rurality			
Urban	17 346	3594 (20.7)	18.0 (17.5-18.6)
Suburban	3003	772 (25.7)	21.6 (20.2-23.0)
Rural	1239	347 (28.0)	24.7 (22.4-26.9)
Patients identifying as White by rurality			
Urban	56 585	9107 (16.1)	16.7 (16.4-17.0)
Suburban	21 175	3627 (17.1)	18.7 (18.1-19.2)
Rural	10 707	1832 (17.1)	18.6 (17.9-19.4)

Abbreviation: NA, not applicable.

a Predicted probabilities are calculated based on the final regression model, which includes all 3 social identities and the interaction term between rural residence and identifying as Black and adjusts for age, sex, whether the patient received Medicaid at any point during the 12 months prior to hospitalization, and comorbidities.

**Table 3.** Social IdentityMetrics for Major Leg Amputation or Death Based on Identifying as Black, Rural Residence, and Neighborhood Disadvantage

Model and variables	Odds ratio (95% CI)			
	Identifying as Black	Rural residence	Living in a disadvantaged neighborhood	Interaction term between rural residence and identifying as Black^a^
Model 1				
Identifying as Black + age + sex	1.60 (1.54-1.66)	NA	NA	NA
Rural residence + age + sex	NA	1.10 (1.05-1.15)	NA	NA
Living in a disadvantaged neighborhood + age + sex	NA	NA	1.25 (1.20-1.29)	NA
Model 2				
Identifying as Black + rural residence + living in a disadvantaged neighborhood + Medicaid + age + sex^b^	1.54 (1.48-1.61)	1.15 (1.09-1.21)	1.10 (1.06-1.14)	NA
Model 3				
Identifying as Black + rural residence + living in a disadvantaged neighborhood + Medicaid + comorbidities + ulcer severity + age + sex^b^	1.15 (1.10-1.20)	1.20 (1.14-1.27)	1.09 (1.05-1.14)	NA
Model 4				
Identifying as Black + rural residence + living in a disadvantaged neighborhood + Medicaid + comorbidities + ulcer severity + age + sex + interaction (identifying as Black × rural residence)^b^	1.11 (1.05-1.16)	1.16 (1.09-1.23)	1.09 (1.05-1.14)	1.34 (1.15-1.57)

Abbreviation: NA, not applicable.

a Only the interaction term between rural residence and identifying as Black is presented because this factor was the sole interaction between the 3 social identitymetrics that was statistically significant.

b Whether the patient received Medicaid coverage in the year prior to hospitalization was used as a marker of patient-level socioeconomic status.

## Key Points

### Question

Is the intersection of race, ethnicity, rurality, and/or neighborhood disadvantage associated with outcomes among US patients with diabetic foot ulcers?

### Findings

In this cohort study of 124 487 patients hospitalized with diabetic foot ulcers, 17.6% underwent major (above-ankle) leg amputation or died within 30 days of hospital discharge; proportions increased to 18.3% for rural patients and 21.9% for those identifying as Black. The proportion increased to 28.0% for patients who identified as both rural and Black, suggesting a role for intersectionality.

### Meaning

This study suggests that the intersection of rural residence and identifying as Black is associated with an amplified risk of major leg amputation or death among US patients with diabetic foot ulcers.
