# Supplementary material for: Association of Race, Ethnicity, and Rurality With Major Leg Amputation or Death Among Medicare Beneficiaries Hospitalized With Diabetic Foot Ulcers
Source: JAMA Netw Open. Author manuscript; Available in PMC 2023 Apr 1. (PMC9024392; doi:10.1001/jamanetworkopen.2022.8399)
Supplement: Online Only Supplement Material — eTable 1. ICD-9 and CPT Procedure Codes Used to Identify Patients Undergoing Major Leg Amputation eTable 2. Odds Ratios for Major Leg Amputation or Death for Main Effects and Interactions Among Social Identities of Interest: Identifying as Black, Rural Residence, and Neighborhood Disadvantage [file NIHMS1800716-supplement-Online_Only_Supplement_Material.pdf]

Supplementary Online Content

Brennan MB, Powell WR, Kaiksow F, et al. Association of race, ethnicity, and rurality with major leg amputation or death among Medicare beneficiaries hospitalized with diabetic foot ulcers. *JAMA Netw Open.* 2022;5(4):e228399. doi:10.1001/jamanetworkopen.2022.8399

eTable 1. *ICD-9* and *CPT* Procedure Codes Used to Identify Patients Undergoing Major Leg Amputation

eTable 2. Odds Ratios for Major Leg Amputation or Death for Main Effects and Interactions Among Social Identities of Interest: Identifying as Black, Rural Residence, and Neighborhood Disadvantage

This supplementary material has been provided by the authors to give readers additional information about their work.

© 2022 Brennan MB et al. *JAMA Network Open.*

eTable 1. *ICD-9* and *CPT* Procedure Codes Used to Identify Patients Undergoing Major Leg Amputation

ICD-9 Procedure Code	*CPT* Code	Description of Procedure
84.13-84.15:<br>Below knee amputation	27880	Amputation leg through tibia and fibula
	27881	Amputation leg through tibia and fibula with immediate fitting technique including application of first cast
	27882	Amputation leg through tibia and fibula, open circulatory (guillotine)
	27886	Amputation leg through tibia and fibula, re-amputation
	27888	Syme/Pirogroff
	27889	Ankle disarticulation
84.16-84.17:<br>Above knee amputation	27295	Disarticulation of hip
	27590	Amputation, thigh, through femur, any level
	27591	Amputation, thigh, through femur, any level, immediate fitting technique including first cast
	27592	Amputation, thigh, through femur, any level, open, circular (guillotine)
	27596	Amputation, thigh, through femur, any level, re-amputation
	27598	Disarticulation at the knee

© 2022 Brennan MB et al. *JAMA Network Open.*

eTable 2. Odds Ratios for Major Leg Amputation or Death for Main Effects and Interactions Among Social Identities of Interest: Identifying as Black, Rural Residence, and Neighborhood Disadvantage

**Model variables**	**Main effects**			**Interactions**			
	**Identifying as Black**	**Rural residence**	**Living in a disadvantaged neighborhood**	**Interaction term between rural residence and identifying as Black**	**Interaction term between rural residence and living in a disadvantaged neighborhood**	**Interaction terms between identifying as Black and living in a disadvantaged neighborhood**	**Interaction term among identifying as Black, rural residence, and living in a disadvantaged neighborhood**
Identifying as Black + rural residence + living in a disadvantaged neighborhood + Medicaid + comorbidities + ulcer severity + age + sex + interaction (identifying as Black x rural residence)[a]	1.11 (1.05-1.16)	1.16 (1.09-1.23)	1.09 (1.05-1.14)	1.34 (1.15-1.57)			
Identifying as Black + rural residence + living in a disadvantaged neighborhood + Medicaid + comorbidities + ulcer severity + age + sex + all interactions among social identities of interest[b]	1.13 (1.07-1.20, p<0.001)	1.13 (1.06-1.22, p<0.001)	1.12 (1.04-1.21, p=0.001)	1.65 (1.33-2.05, p<0.001)	1.03 (0.90-1.19, p=0.592)	0.92 (0.82-1.02, p=0.142)	0.70 (0.51-0.96, p=0.025)

[a]This is the final, Model 4, presented in the main text.
[b]This is the penultimate model that we built to check for significant interactions between our social identities of interest. It included all possible interactions. From there, we winnowed down to Model 4 by including only significant interaction terms, which tightened the confidence intervals.

© 2022 Brennan MB et al. *JAMA Network Open.*
